# The Preperitoneal Space in Hernia Repair

**DOI:** 10.3389/fsurg.2022.869731

**Published:** 2022-05-30

**Authors:** A. Lorenz, C. Augustin, M. Konschake, P. Gehwolf, B. Henninger, F. Augustin, D. Öfner

**Affiliations:** ^1^ Department of Visceral, Transplant and Thoracic Surgery, Medical University of Innsbruck, Innsbruck, Austria; ^2^ Department of Anatomy, Histology and Embryology, Institute of Clinical and Functional Anatomy, Medical University Innsbruck (MUI), Innsbruck, Austria; ^3^ Department of Radiology, Innsbruck Medical University, Innsbruck, Austria

**Keywords:** abdominal wall repair, groin hernia, retroperitoneum, anatomy, transversalis fascia

## Abstract

The preperitoneal spaces relevant for incisional hernia repair and minimally invasive groin hernia repair are described in terms of surgical anatomy. Emphasis is put on the transversalis fascia and the urogenital fascia and its extensions, the vesicoumbilical fascia, and the spermatic sheath of Stoppa procedure. Steps in hernia surgery where these structures are relevant are reviewed.

## Introduction

Recognizing and dissecting along the anatomical planes is the (hernia) surgeons’ pleasure; failing to do so puts the patient at risk for postoperative pain and prolonged operative and recovery times. To a surgeon familiar with operating inside the peritoneal cavity, the peritoneum and the preperitoneal space can pose a surprising challenge when pursuing it from the outside. This situation occurs during hernia repair with mesh augmentation at the junction of the abdomen and the pelvis, typically total extraperitoneal laparoscopic hernioplasty, Stoppa procedure, or a transversus abdominis muscle release (TAR) maneuver. Its success depends on understanding how the retroperitoneal structures from the abdomen enter the pelvis alongside the peritoneal sac.

## What is the Preperitoneal Space?

The preperitoneal space is commonly described as the space between the peritoneum and transversalis fascia (TF). It is also referred to as extraperitoneal, preperitoneal, or retroperitoneal space, emphasizing that it is not limited to the ventral portion of the abdomen.

While the peritoneum is readily identified, the TF is described as a complex entity with a bilaminar structure that covers the muscles that surround the abdominal cavity. In 1804, Cooper (1) in his work on inguinal and femoral hernia and without using the term TF noted, “A thinner fascia is sent upward from the crural arch immediately behind the abdominal muscles, to which it gives a lining similar to that tendinous expansion which covers them on the fore part. This is the fascia which leaves an opening from the abdomen for the spermatic cord in the male, and for the round ligament of the uterus in the female.” In the second edition of his textbook in 1844, he named this structure the transversalis fascia. Publications by Cruveilhier (2), Mackay (3), McVay and Anson (4), and Lytle (5) helped to perpetuate the notion of this fascia and introduced the concept of a bilaminar structure with a deep and a superficial layer (or anterior and posterior lamina). This bilaminar arrangement described by surgeons (6) is neither reproduced in contemporary textbooks of anatomy nor by *Terminologia Anatomica* (7). *Gray’s Anatomy* (8) describes the TF as follows: “The transversalis fascia is a thin stratum of connective tissue lying between the internal surface of the transversus [muscle] and the extraperitoneal fat. It is part of the general layer of fascia between the peritoneum and the abdominal walls, and is continuous with the iliac and pelvic fasciae. The spermatic cord in the male, or the round ligament of the uterus in the female, pass through the transversalis fascia at the deep inguinal ring.” This general layer of fascia between the peritoneum and the abdominal wall is also referred to as the parietal abdominal fascia or the endo-abdominal fascia (*Terminologia Anatomica*) (9).

**Figure 1:**
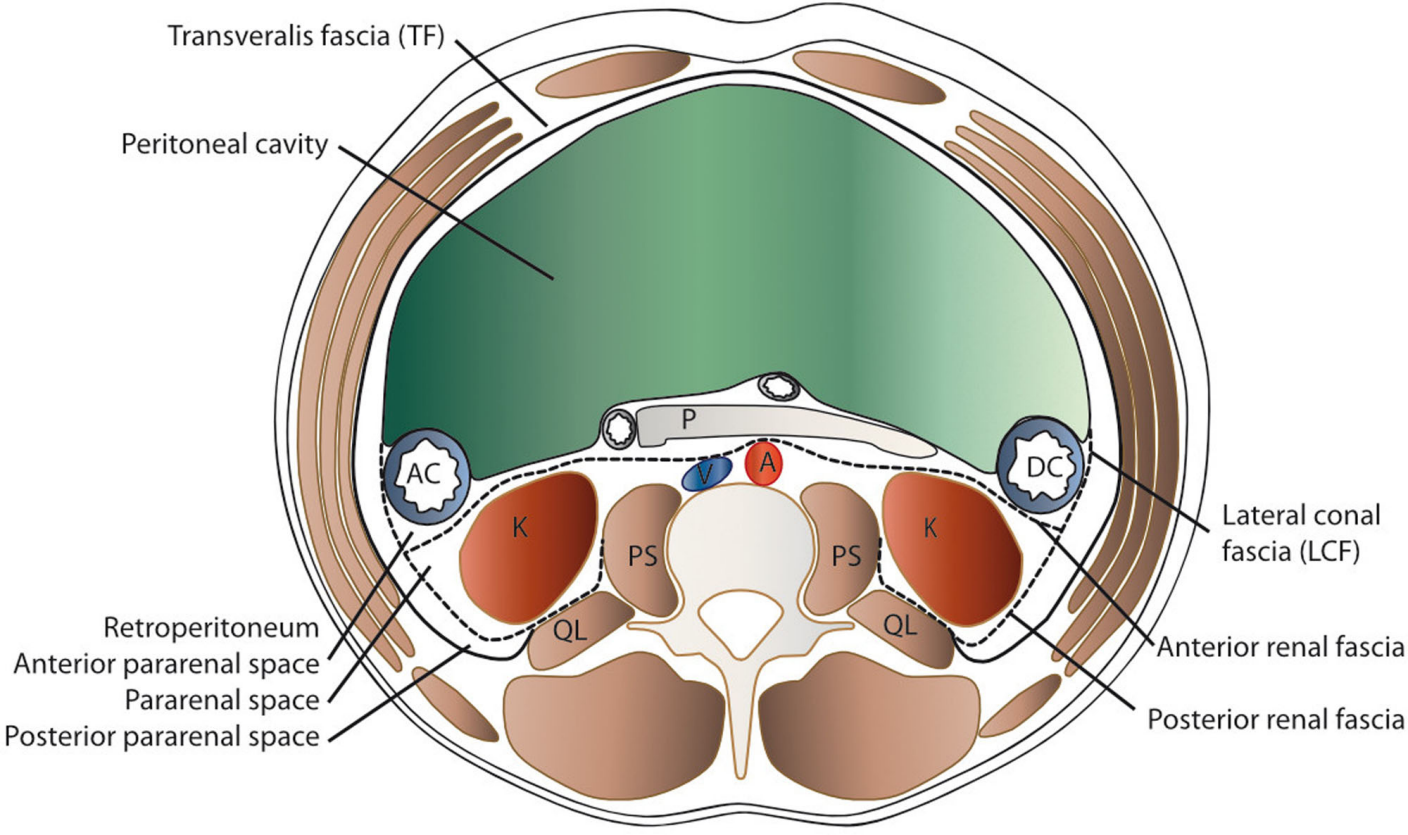
Schematic transverse section of the abdomen with emphasis on the extraperitoneal spaces. The abdominal structures are separated from the musculoskeletal structures by the transversalis fascia (TF). The peritoneal cavity is delimited by the parietal peritoneum, which is the anterior border of the anterior pararenal space (APS) that contains the ascending (AC) and descending (DC) colon, pancreas (P), and duodenum (D). Posteriorly lies the perirenal space (PRS) with the kidney (K) and medially the aorta (A) and vena cava (V), delimited by the anterior renal fascia (anterior RF or Gerota’s fascia) and the posterior renal fascia (posterior RF or Zuckerkandl’s) and laterally the lateroconal fascia (LCF). Dorsally, the quadratus lumborum (QL) and psoas major (PS) muscles are found within the spine.

**Figure 2:**
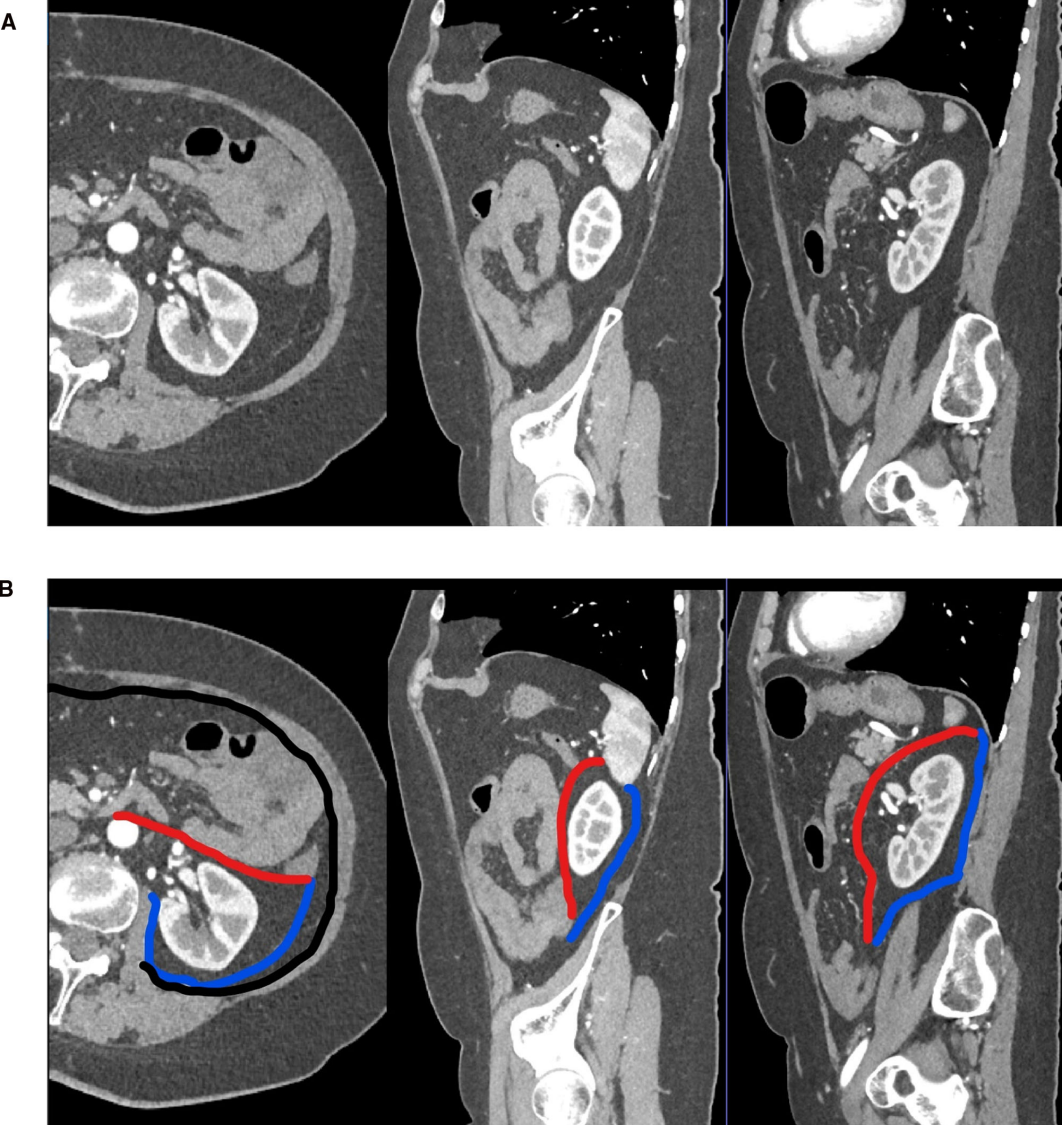
Computed tomography imaging with a reconstruction of the transverse and sagittal planes with contrast enhancement (**A**) and emphasizing the perirenal fat pad and its limits. (**B**) Same images with the posterior renal fascia highlighted in blue, the anterior renal fascia in red, and TF in black.

The contents of the preperitoneal space are as complex as its limits (Figure 1). Ignoring the question of whether the TF is a separate entity or an extension of its muscle (10), the space between it and the peritoneum can be interpreted as a fat padded pathway for the gonads and kidneys that traverse it during embryonic development and for vessels and nerves to the lower extremity. Accordingly, the perirenal fat envelops the kidneys and extends into the pelvis, where the kidneys originate from the metanephrons and are tethered to the urinary bladder by the ureter. This configuration is similar to an inverted cone, with the tip in the pelvis and the diaphragm at the base (Figure 2) and is referred to as the perirenal or urogenital fascia. The anterior margin is the anterior renal fascia or Gerota’s fascia, and the posterior margin is the posterior renal fascia or Zuckerkandl’s fascia, which laterally extends caudally into the lateroconal fascia [debated by Marks et al. (11) and Raptopoulos et al. (12)]. Whether the tip of the cone is closed by fusion of the lateroconal fascia to the peritoneum or it prolongates into the preperitoneal space in the iliac fossa is still a matter of discussion (13). Between this cone defined by the perirenal fascia and the TF lies a layer of fat dorsally and laterally. A depiction of these structures as they extend from the kidneys to the pelvis is given in Supplementary Figure S1.

**Figure 3:**
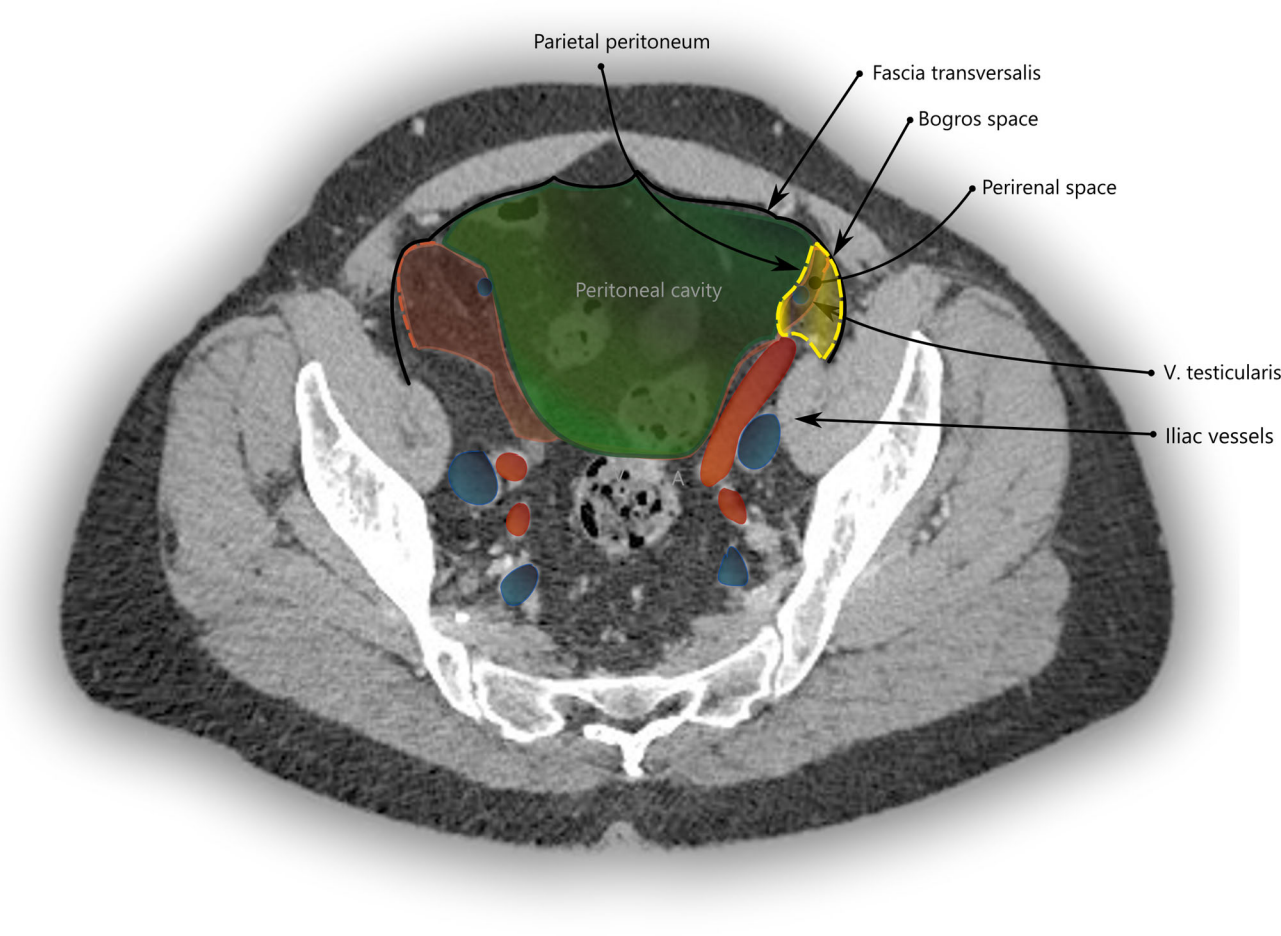
CT imaging with a transverse section through the pelvis at the apex of the urinary bladder. The space of Bogros is marked in yellow and limited by the TF (black) laterally and the parietal peritoneum (green) medially. It contains the extension of the perirenal space (red) into the pelvis.

In the iliac fossa, a triangular space enclosed by the TF anteriorly, the parietal peritoneum medially and laterally by the iliac fascia is referred to as the space of Bogros (14) (Annet Jean Bogros 1786–1825, a French anatomist and surgeon). The iliac fascia, as quoted above from *Gray’s anatomy*, is continuous with the TF. As is visualized in Figure 3 and in more detail in Supplementary Figure S1, the space of Bogros contains the extension of the perirenal fascia into the pelvis.

Medially, the space between the pubic symphysis and the urinary bladder is called the retropubic space or the space of Retzius (15) (Anders Retzius 1796–1860, a Swedish anatomist). It lies in front and to the sides of the urinary bladder.

In the context of hernia surgery, the space of Bogros and the space of Retzius were popularized by Bendavid, stating that the former is an extension of the latter (16). This is debated by Kingsnorth et al. (17) among others, who describe the retropubic space extending laterally to a plane that holds the epigastric vessels and lies ventral of the TF. This is relevant when extending surgical dissection from the space of Retzius to the space of Bogros or vice versa. For details, see Hureau (18).

The routine use of computed tomography (CT) has widened our understanding of the preperitoneal space considerably, as it has provided a large sample size of fluid and gas dissemination along constant pathways or tissue planes in retroperitoneal diseases such as pancreatitis, bleeding, cancer, or duodenal perforation (19). This is replicated by CT studies on cadavers, where radio-opaque fluids have been instilled experimentally in different parts of the preperitoneal space (20).

## How do we Reach the Preperitoneal Space: Transabdominal Approach, Total Extraperitoneal Approach, and Transversus Abdominis Release

In minimal invasive inguinal hernia repair, the transabdominal approach (TAPP) takes the direct path to the preperitoneal space by incising the peritoneum from within the peritoneal cavity and peeling it away from the structures that it adheres to.

**Figure 4:**
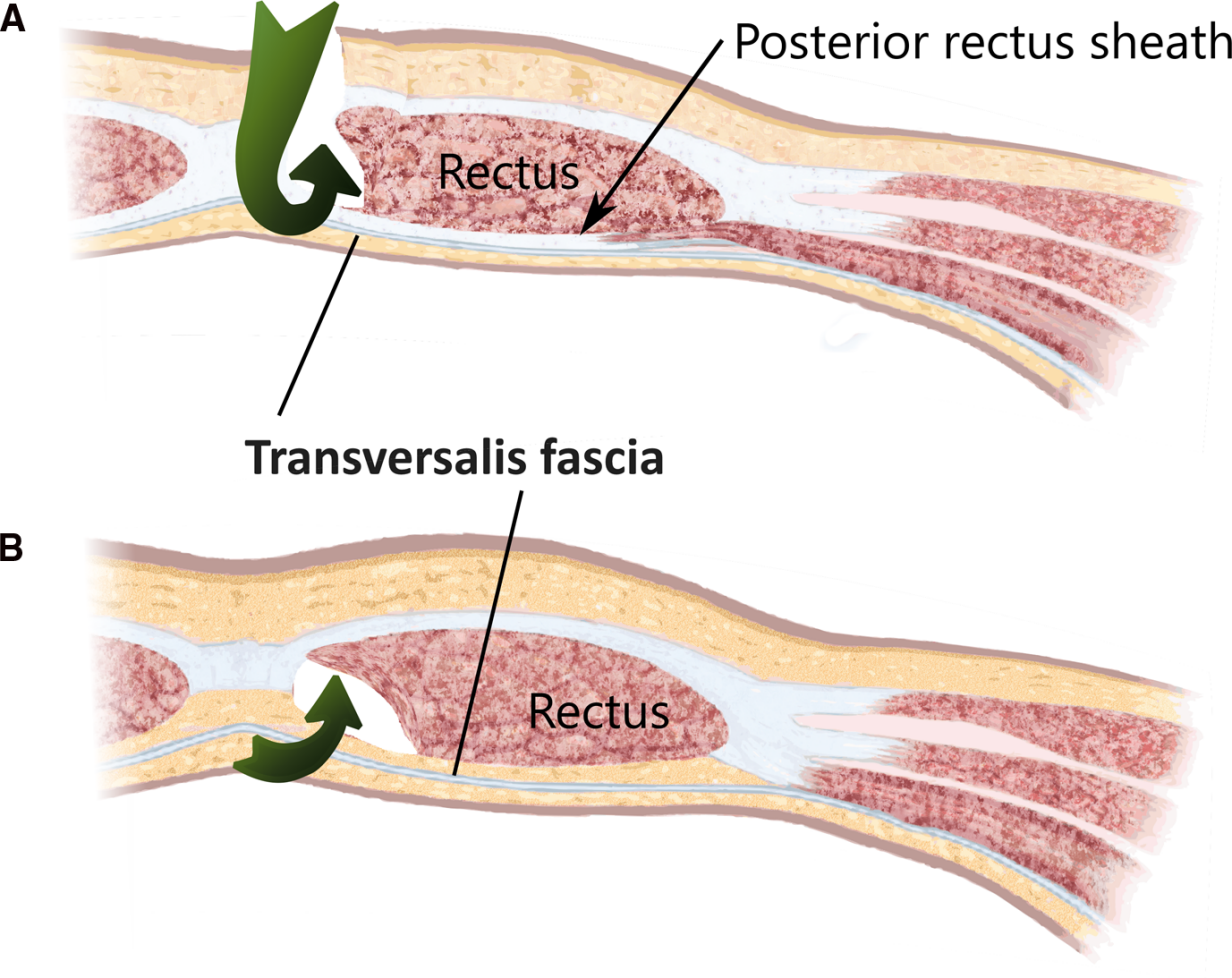
The surgical access (green arrows) of a total extraperitonal hernioplasty through the layers of the ventral abdominal wall are depicted (**A**) cranial and (**B**) caudal of the arcuate area. The transversalis fascia passes the posterior rectus sheath dorsally and continues beyond the arcuate area and separates the musculoaponeurotic structures from the visceral peritoneum.

**Figure 5:**
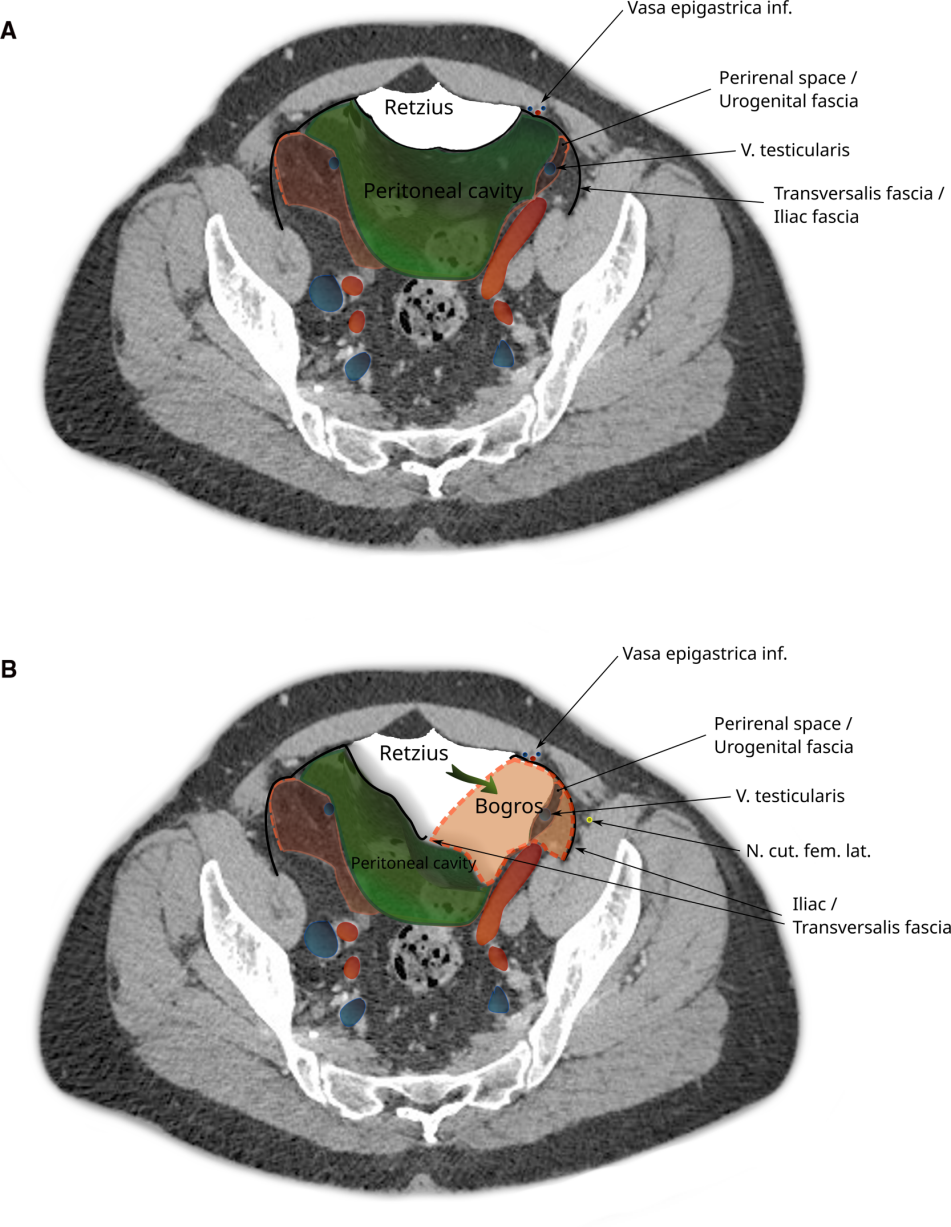
CT image of the pelvis showing dissection during a total extraperitoneal hernioplasty (TEP). (**A**) The peritoneal sac is displaced and the space of Retzius dissected. (**B**) The space of Bogros is opened by cleaving the transversalis fascia. Note that the space of Bogros contains the spermatic vessels and tissues from the perirenal space enveloped by the urogenital fascia that extends from the abdomen into the pelvis.

The total extraperitoneal approach (TEP) requires dissecting through the transveralis fascia in order to reach the preperitoneal space. By typically entering through an umbilical incision into the rectus sheath and following the retromuscular plane, the surgeon is left external to the TF. While the transverse abdominal muscle inserts aponeurotically into the rectus sheath, its fascia is described to pass posterior to the rectus muscle and linea alba (Figure 4A) and continues beyond the arcuate area where the dissection during TEP starts (Figure 4B). In most cases, the TF here is already ruptured by blunt dissection with a pneumatic dissector balloon or a laparoscopic camera at this point of the TEP procedure and the space of Retzius (Figure 5A) is opened (this can be felt as a loss of resistance when advancing the dissection balloon toward the symphysis). Often, the fibers of the arcuate area can be seen to extend toward the urinary bladder medially and the myopectineal orifice laterally. This has been described as the umbilical-prevesical fascia (or vesicoumbilical fascia), which itself is thought to be an extension of the urogenital fascia (or perirenal fascia) (21). When dissecting laterally, in order to find the peritoneal sac (and reduce the hernia), this extension of the urogenital fascia has to be transected. This step of dissection (Figure 5B) is referred to as a switch between a superficial parietal layer (anterior sub-peritoneal fascia) and a deep visceral layer (posterior sub-peritoneal fascia) by some (22), whereas Stoppa procedure described a “spermatic sheath,” a triangular fibrocellular lamella sheathing the spermatic cords and gonadal vessels in males and a similar genital sheath in females (23). This extension of the urogenital fascia divides the space of Bogros into a cleavable space between the peritoneum and genital sheath without notable vasculature and a space between the TF and the genital sheath containing nerves and blood vessels. Incidentally, this extension of the urogenital fascia to the myopectineal orifice at times is found to contain fatty tissue herniating through the inguinal ring, presenting as a spermatic cord lipoma.

Finally, during (incisional) ventral hernia repair, the dissection from the retrorectus plane (24) can be extended laterally by the TAR maneuver with posterior component separation. Here, the posterior rectus sheath laterally is transected together with the underlying transverse abdominal muscle or its aponeurosis (depending on how cranial or caudal this step is performed), and by transecting the TF, the parietal peritoneum is exposed.

## How do We Miss the Preperitoneal Space: Pitfalls During Total Extraperitoneal Approach, Transabdominal Approach, Stoppa Procedure, and Transversus Abdominis Release Procedures

**Figure 6:**
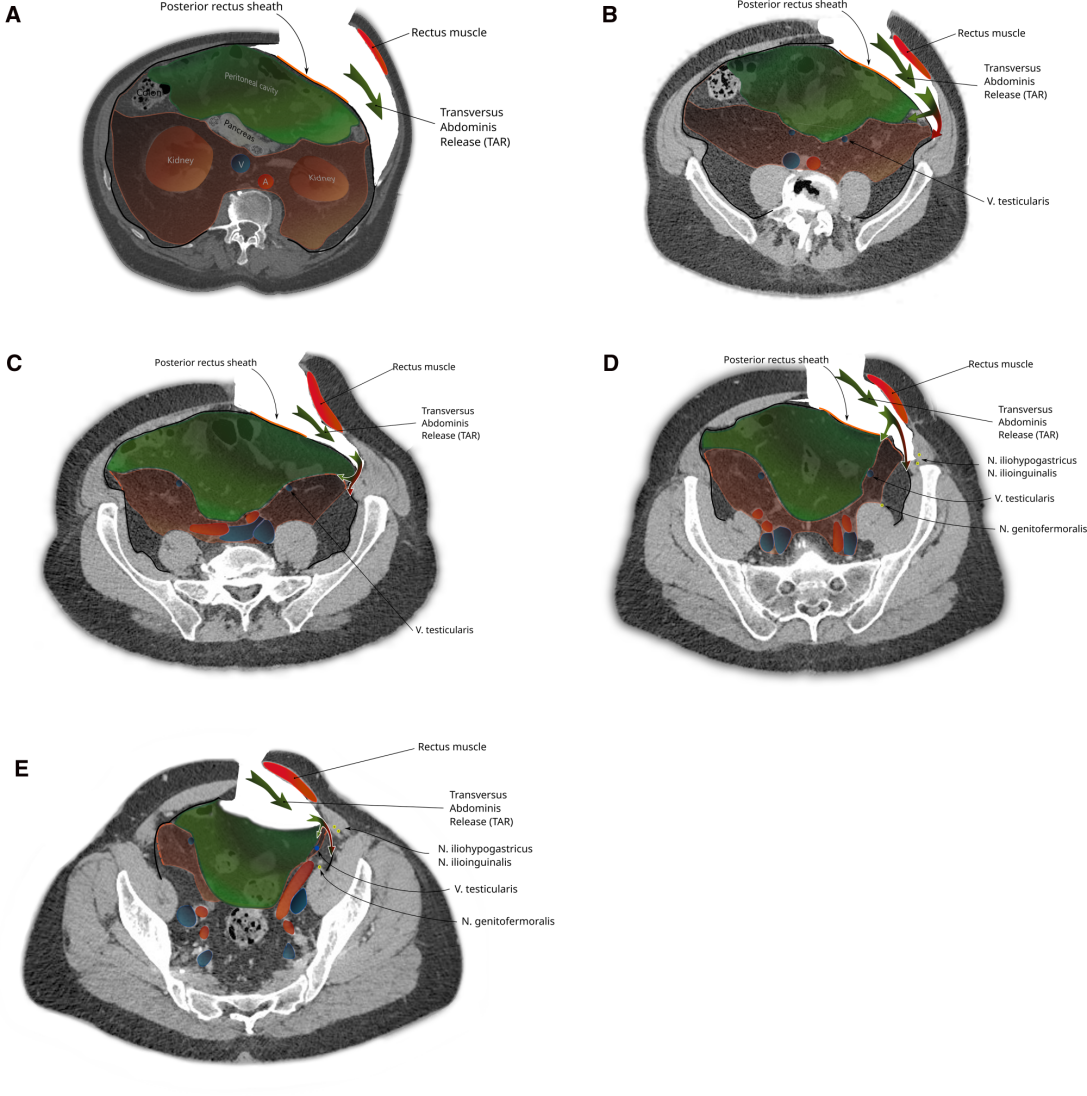
The steps of a transversus abdominis release-maneuver (TAR): (A) Lateral incision of the posterior rectus sheath and dorsolateral dissection along the transverse abdominis muscle (green arrow). Caudal extension of the dissection follows the urogenital fascia into the pelvis (B-E) reaching the space of Bogros in the superficial parietal layer that is so carefully avoided during a TEP procedure (red part of bifurcated arrow). A switch of dissection into the deep visceral layer, analogue to the dissection in TEP is necessary if a prosthetic mesh should also cover a groin hernia (green part of bifurcated arrow).

During a TAR with posterior component separation, when the TF is stripped from its muscle in order to establish a retromuscular (24) mesh position, the preperitoneal space is missed, especially in cases of extended posterior-lateral dissection (as for lumbar incisional hernias). When not following the peritoneal sac, dissection leads behind the kidney and exposes the iliohypogastric and ilioinguinal nerves (Figure 6).

**Figure 7:**
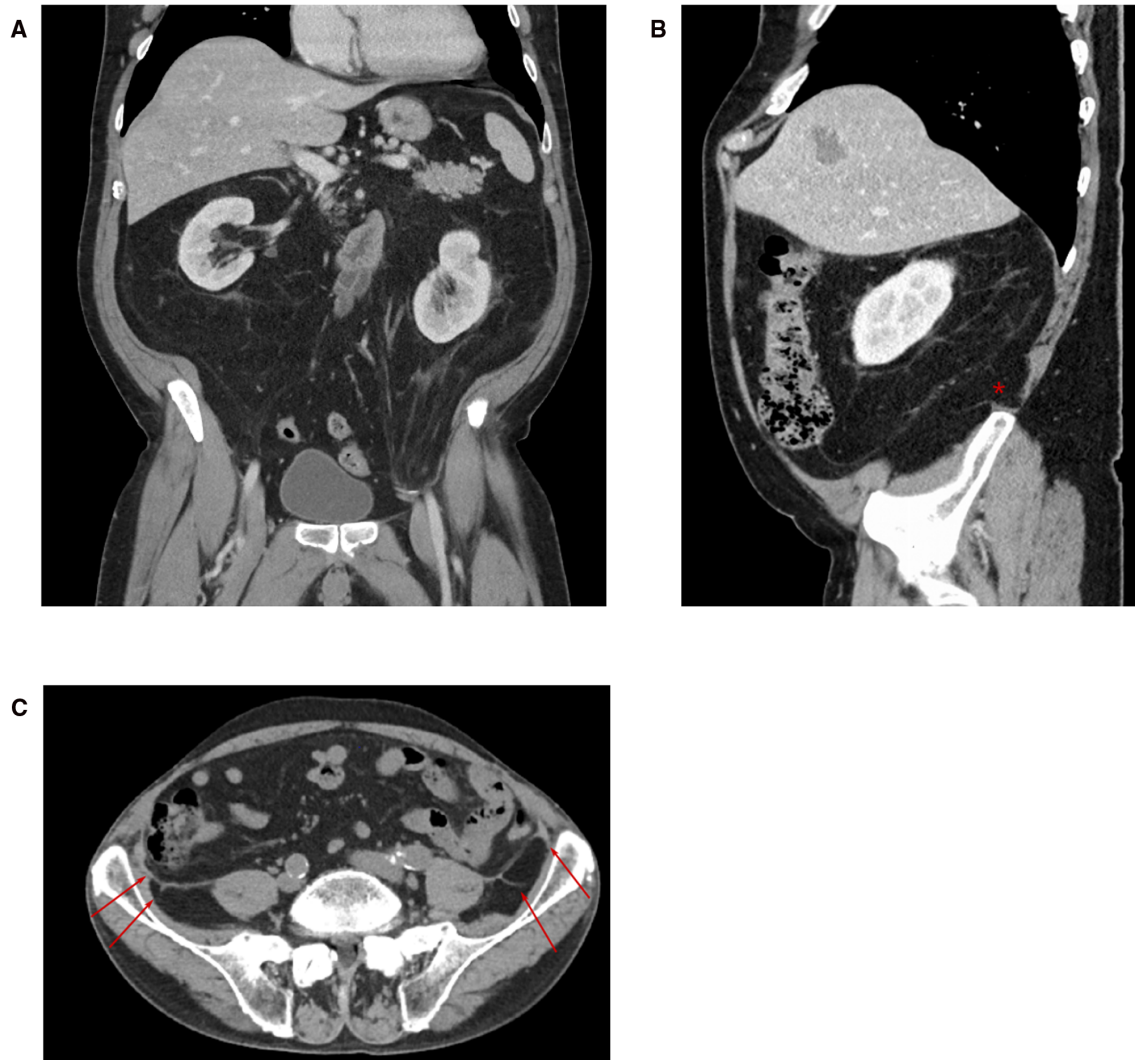
CT imaging with coronal (**A**) and sagittal (**B**) sections through the abdomen. The pararenal fat extends into the pelvis and in this patient ultimately into the left inguinal canal like an inverted cone. An attachment of the posterior renal fascia to the transversalis fascia at the ililac crest can be seen in the sagittal section (*) and (**C**) transverse section (arrows).

When the dissection is started more cranially (as in a classical top to bottom TAR) and extended into the pelvis (e.g., repair of EHS M4 and M5 hernia), the urogenital fascia (posterior renal fascia, the lateroconal fascia) regularly is found attached to the TF with blood vessels (muscular branches of the deep circumflex iliac vessels) crossing at the height of the iliac crest (Figure 7). Division of these attachments and attempting to reach the space of Bogros carry the risk of exposing the genitofemoral nerve and the lateral cutaneous femoral nerves by dissecting the TF off the pelvic muscles (as the TF is in continuation with the iliac and pelvic fascia).

The Madrid modification (down to up) of the TAR procedure starts dissection caudal, as the lower two-thirds of the lateral abdomen contain preperitoneal fat that protect the parietal peritoneum during transection of the aponeurosis of the transversus abdominis muscle and the TF (25). Upon reaching the superior third of the lateral abdomen, this fatty layer wanes and the dissection is continued on purpose external of the TF, which eventually becomes the diaphragmatica fascia.

During inguinal hernia repair by TEP or Stoppa’s procedure, the dissection progresses from medial to lateral of the myopectineal orifice (crossing the testicular vein or the round ligament). At this point, failing to divide the adherent fibers of the urogenital fascia to the inguinal ring (the spermatic sheath as described by Stoppa procedure) and neglecting to switch between the superficial parietal layer (anterior sub-peritoneal fascia) and deep visceral layer (posterior sub-peritoneal fascia), the dissection exposes the iliac muscle and the lateral cutaneous femoral nerve. In abstract terms, this happens because the space of Retzius and the space of Bogros are not in the continuation of each other and are in different planes in relation to the peritoneum (26).

During a TAPP procedure, this is avoided by dissecting from lateral to medial, starting in the correct plane of the preperitoneal space, facilitated by pneumodissection. During TEP, hoping to avoid a peritoneal injury with consequent loss of pre-pneumoperitoneum, the surgeon tends to stay away from the peritoneum when dissecting lateral of the myopectineal orifice and strays laterodorsal of the urogenital fascia risking nerve injury.

## Conclusion

The widespread adoption of minimally invasive techniques for groin hernia surgery and the advent of component separation techniques in ventral incisional hernia repair raise the interest in the anatomic study of preperitoneal spaces and fasciae. Advances in radiology contribute to the beneficial exchanges between anatomy and surgery.

## Author Contributions

AL: conceived the study and drafted the manuscript. CA: extended the manuscript and critically revised it. MK: critically revised the manuscript. PG: critically revised the manuscript. BH: critically revised the manuscript and contributed to radiologic imaging. FA: critically revised the manuscript and designed figures. DÖ: critically revised the manuscript and provided funding for publication fees. All authors contributed to the article and approved the submitted version.

## Supplementary Material

The Supplementary Material for this article can be found online at: https://www.frontiersin.org/articles/10.3389/fsurg.2022.869731/full#supplementary-material.




