# Supplementary material for: The Preperitoneal Space in Hernia Repair
Source: Front Surg. 2022 May 30;9:869731. doi: 10.3389/fsurg.2022.869731 (PMC9197412; doi:10.3389/fsurg.2022.869731)
Supplement: Supplementary file 1 [file Table_2_v1_1.docx]

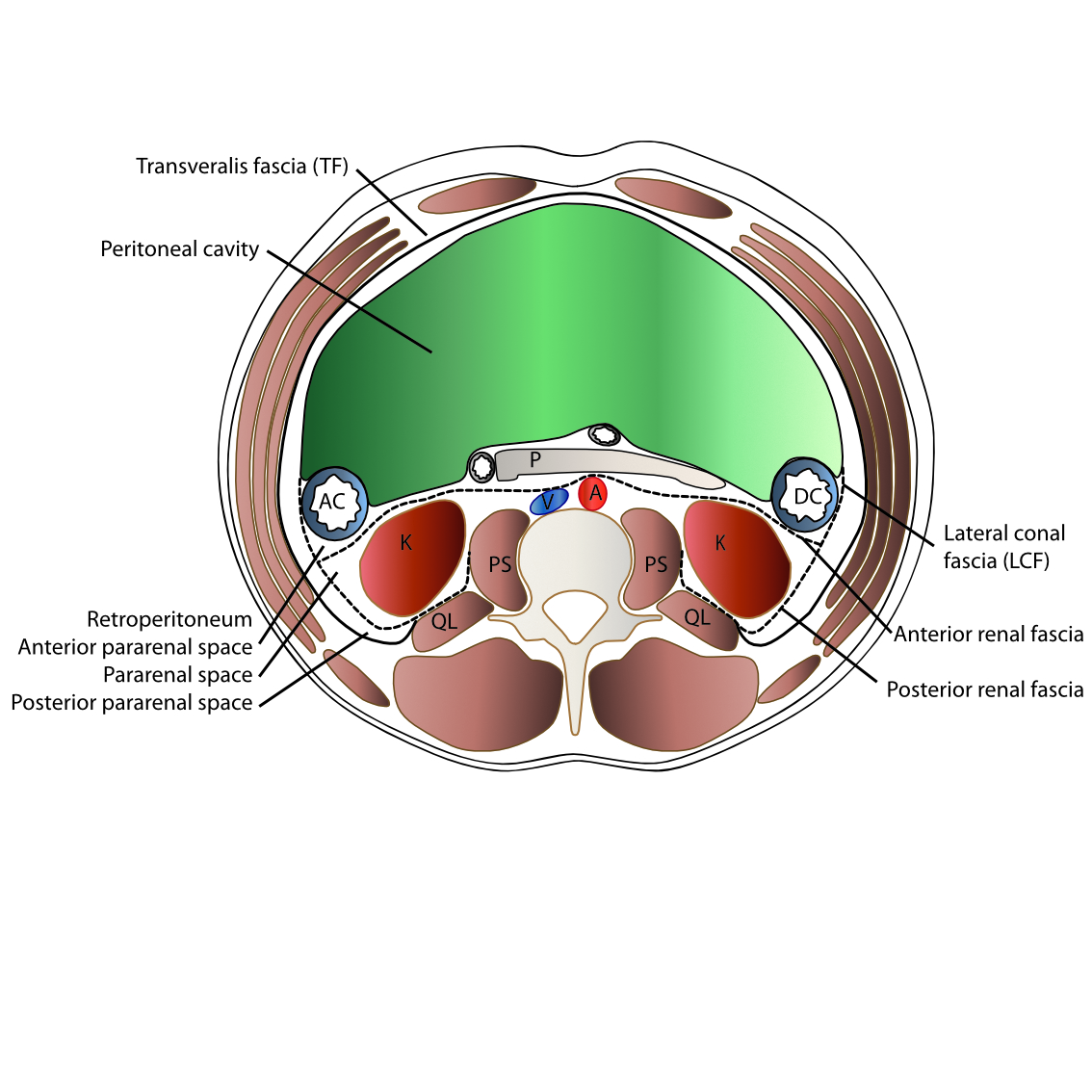


Fig 1. A schematic transverse section of the abdomen with emphasis on the extraperitoneal spaces. The abdominal structures are separated from the musculoskeletal structures by the transversalis fascia (TF). The peritoneal cavity is delimited by the parietal peritoneum, which is the anterior border of the anterior pararenal space (APS) that contains the ascending (AC) und descending (DC) colon, pancreas (P) and duodenum (D). Posteriorly lies the perirenal space (PRS) with the kidney (K) and medially the aorta (A) and vena cava (V), delimited by the anterior renal fascia (anterior RF or Gerota’s fascia) and the posterior renal fascia (posterior RF or Zuckerkandl’s) and laterally the lateroconal fascia (LCF). Dorsally, the quadratus lumborum (QL) and psoas major (PS) muscles are found with the spine.

A
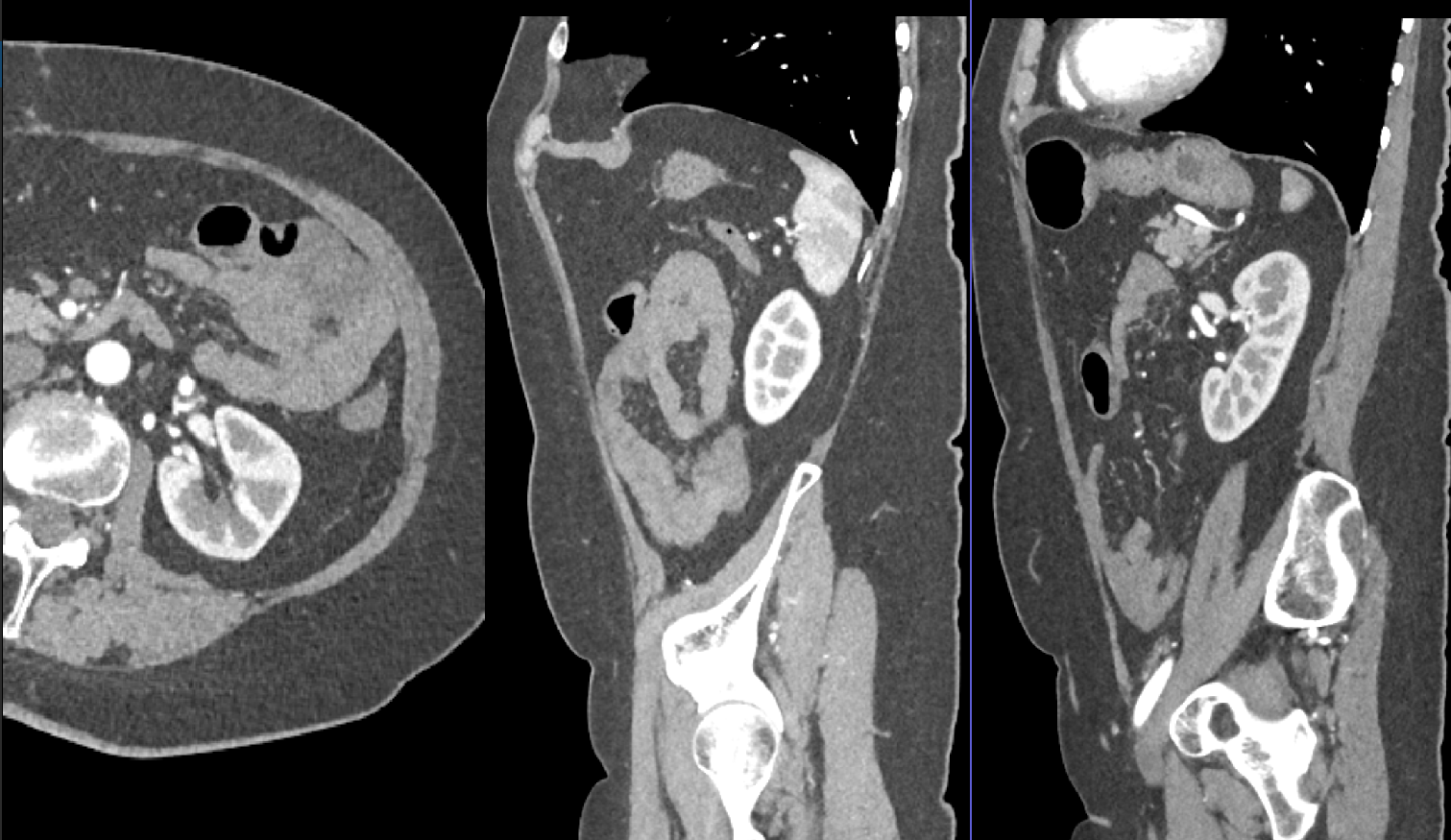


B
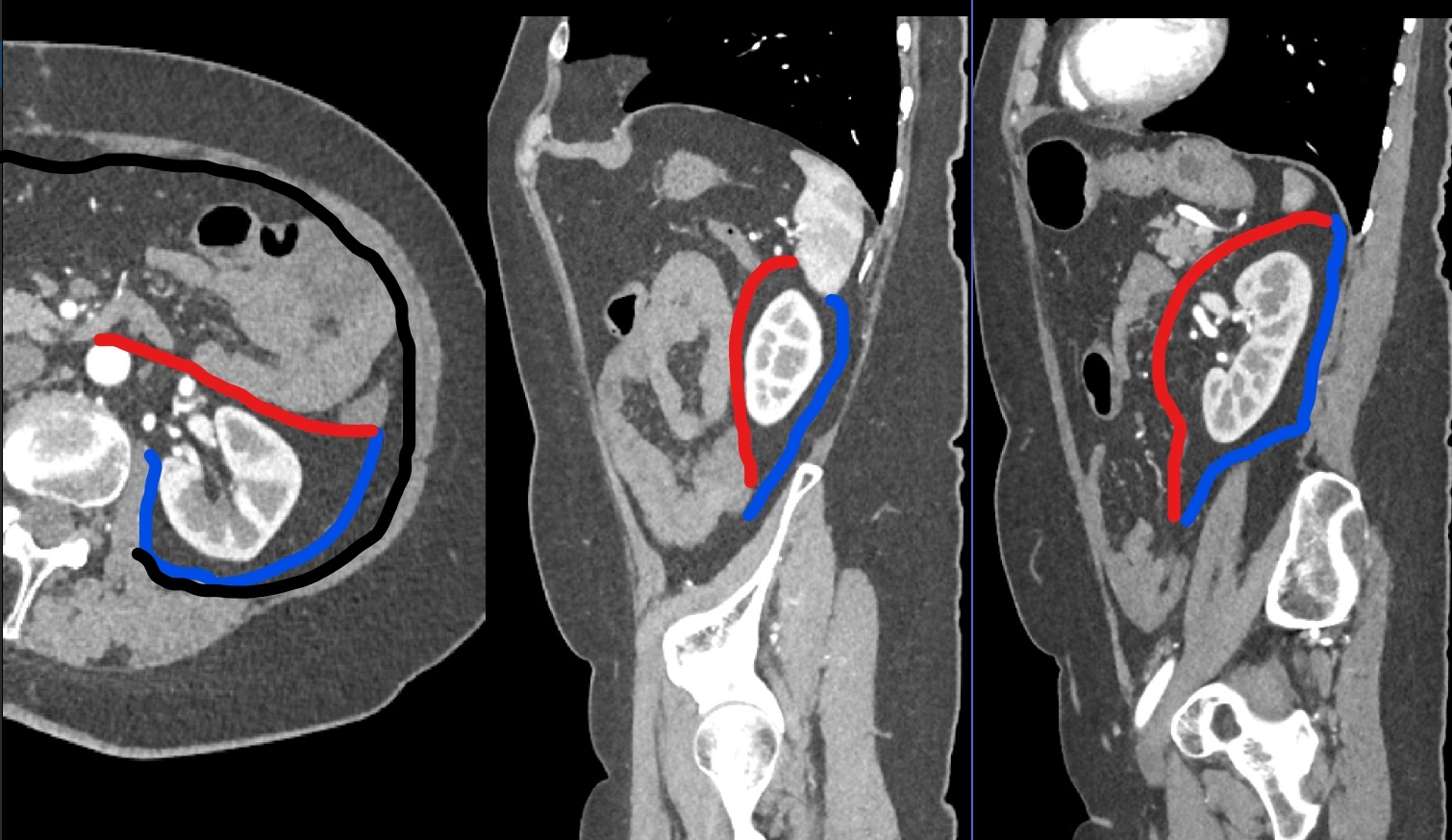


Fig 2. CT imaging with reconstruction of the transverse and sagittal planes with contrast enhancement (A) and emphasizing the perirenal fat pad and its limits. (B) Same images with the posterior renal fascia highlighted in blue, the anterior renal fascia in red and transversalis fascia in black.


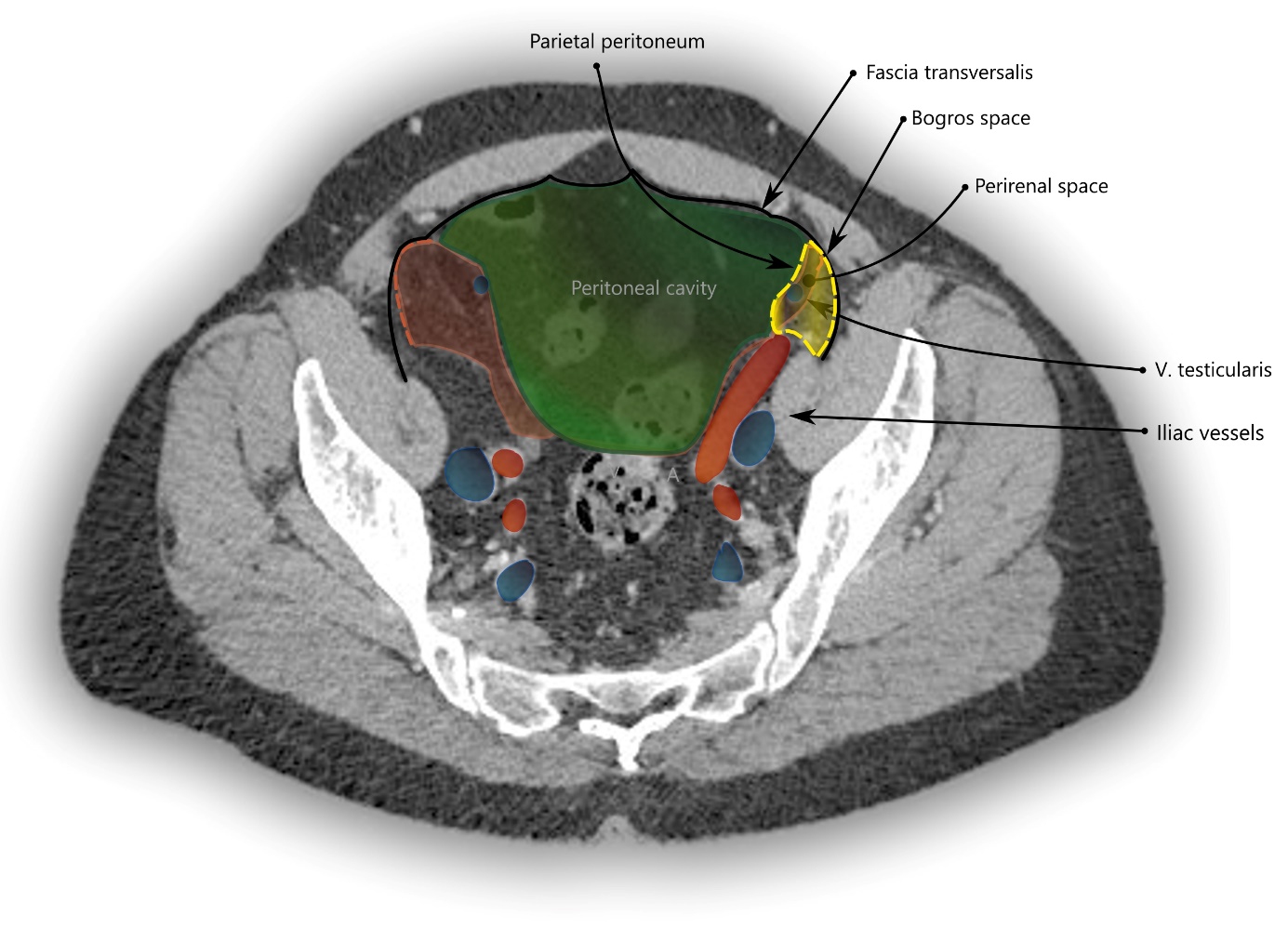


Fig 3. CT imaging with transverse section through the pelvis at the apex of the urinary bladder The space of Bogros is marked in yellow and limited by the transversalis fascia (black) laterally and the parietal peritoneum (green) medially. It contains the extension of the perirenal space (red) into the pelvis.


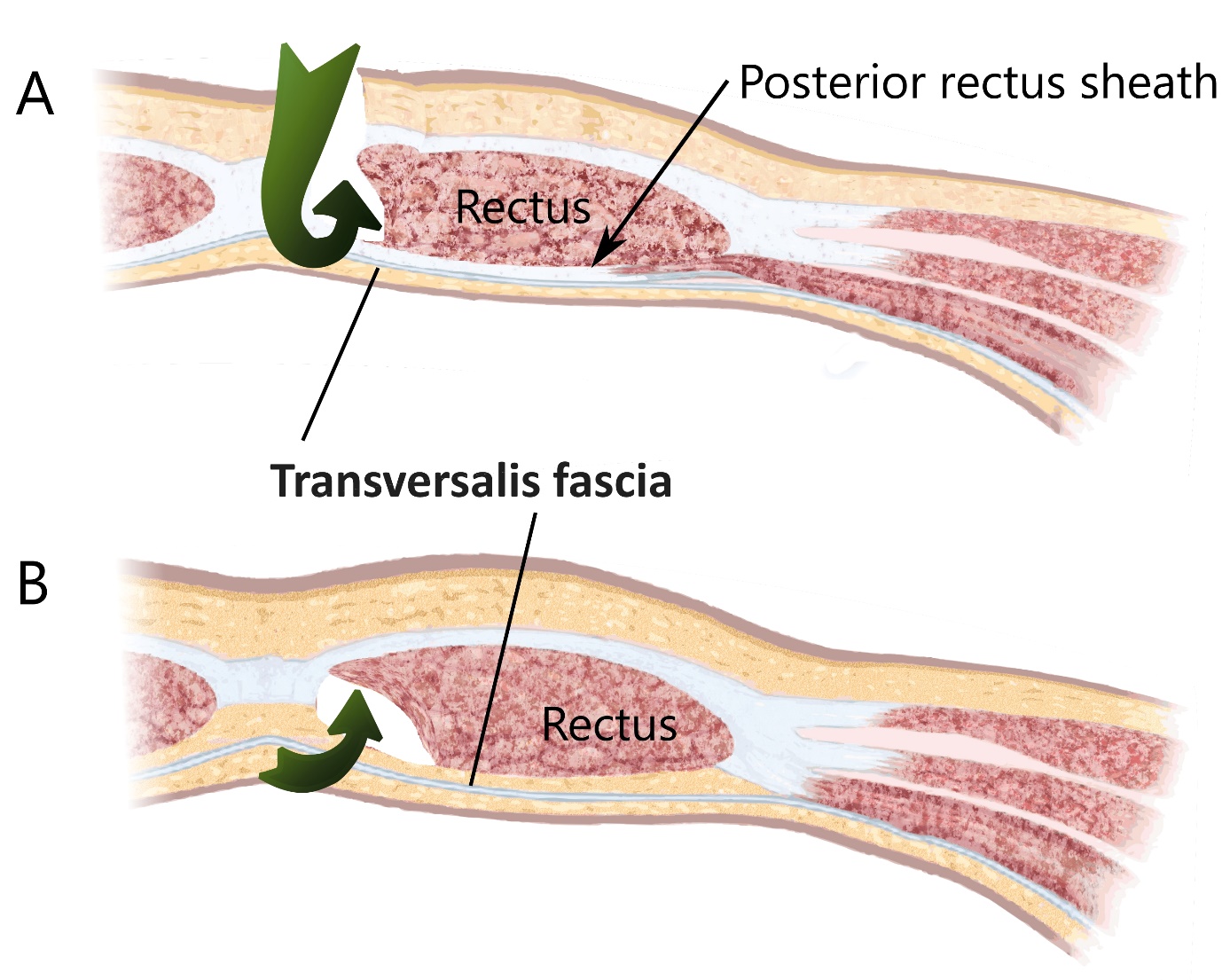


Fig 4. The layers of ventral abdominal wall A) cranial and B) caudal of the arcuate area with the transveralis fascia limiting the musculoaponeurotic structures dorsally. The green arrows indicate the surgical dissection during a total extraperitoneal patch plasty (TEP).


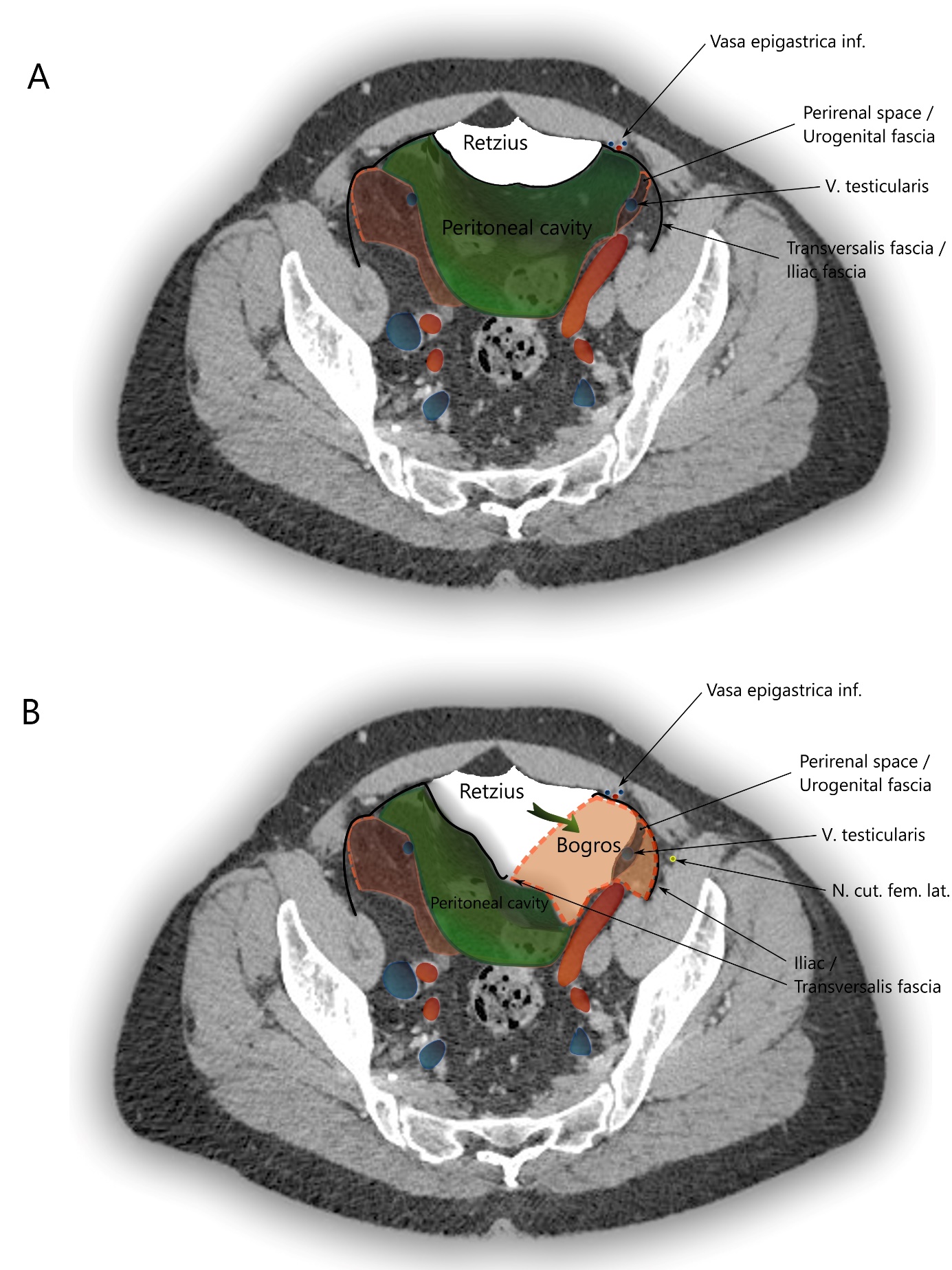


Fig 5. The dissection during a TEP procedure is shown starting in the space of Retzius (A), switching the layer of dissection from superficial parietal to deep visceral and reaching the space of Bogros (B).


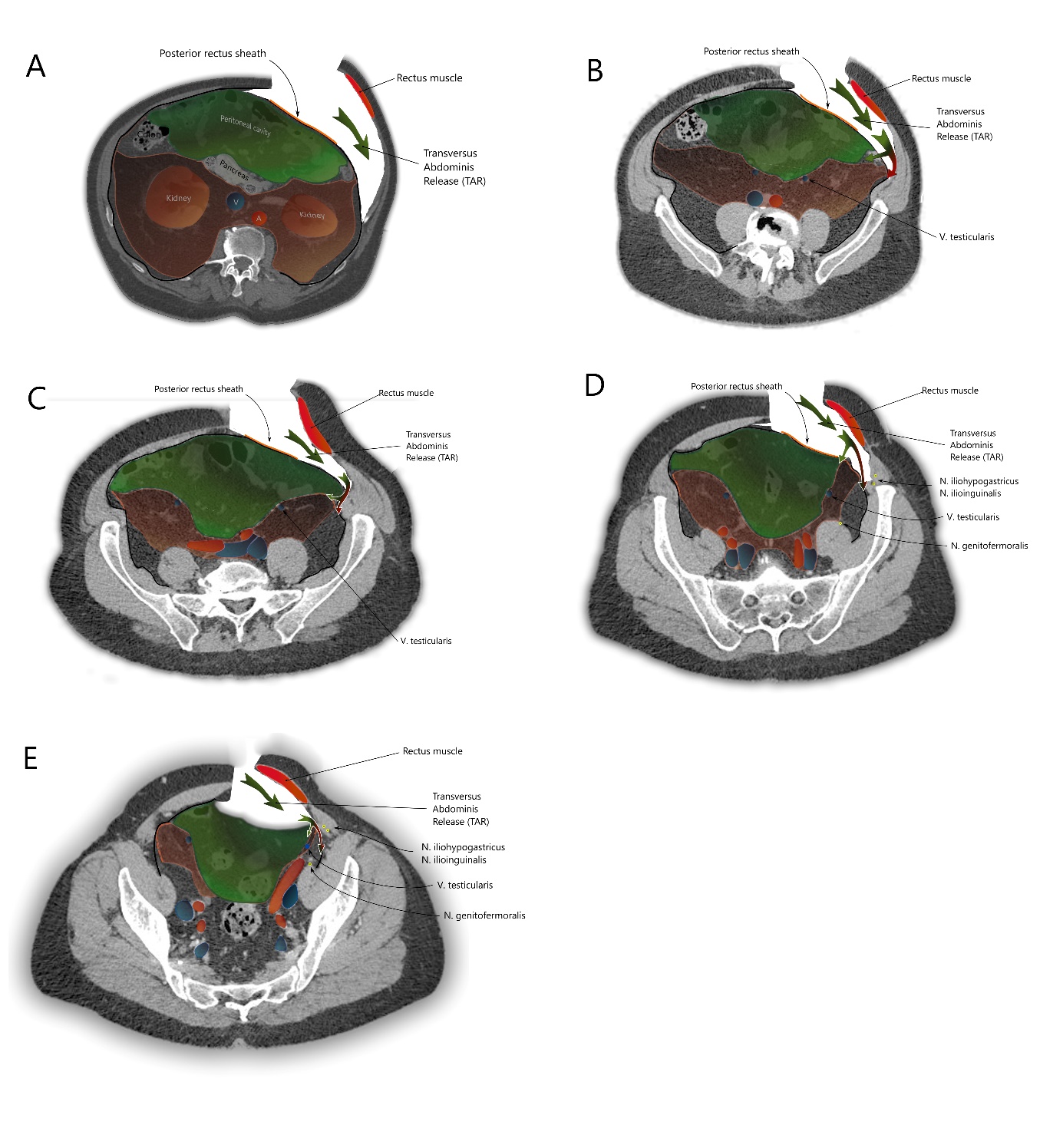


Fig 6. The steps of a transversus abdominis release-maneuver (TAR): (A) Lateral incision of the posterior rectus sheath and dorsolateral dissection along the transverse abdominis muscle (green arrow). Caudal extension of the dissection follows the urogenital fascia into the pelvis (B-E) reaching the space of Bogros in the superficial parietal layer that is so carefully avoided during a TEP procedure (red part of bifurcated arrow). A switch of dissection into the deep visceral layer, analogue to the dissection in TEP is necessary if a prosthetic mesh should also cover a groin hernia (green part of bifurcated arrow).


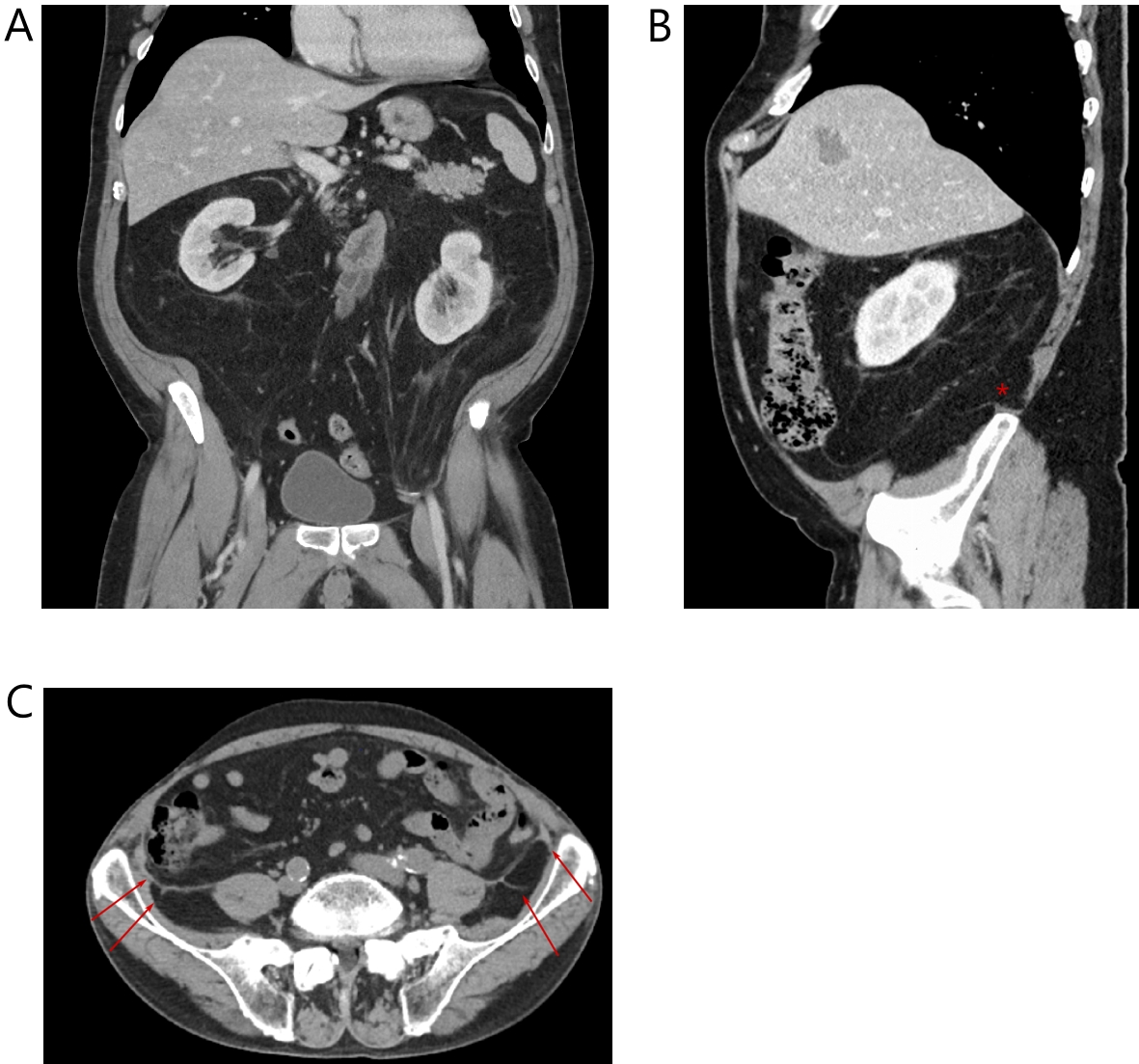


Fig 7. CT imaging with coronal (A) and sagittal (B) sections through the abdomen. The pararenal fat extends into the pelvis and in this patient ultimately into the left inguinal canal like an inverted cone. An attachment of the posterior renal fascia to the transversali fascia at the ililac crest can be seen in the sagittal section (*). (C) A transverse section at the level of the anterior superior iliac spine. The posterior renal fascia appears to be attached to the fascia transversalis laterally (arrows).
