# Supplementary material for: The Preperitoneal Space in Hernia Repair
Source: Front Surg. 2022 May 30;9:869731. doi: 10.3389/fsurg.2022.869731 (PMC9197412; doi:10.3389/fsurg.2022.869731)
Supplement: Supplementary file 2 [file Table_2_v1_2.docx]

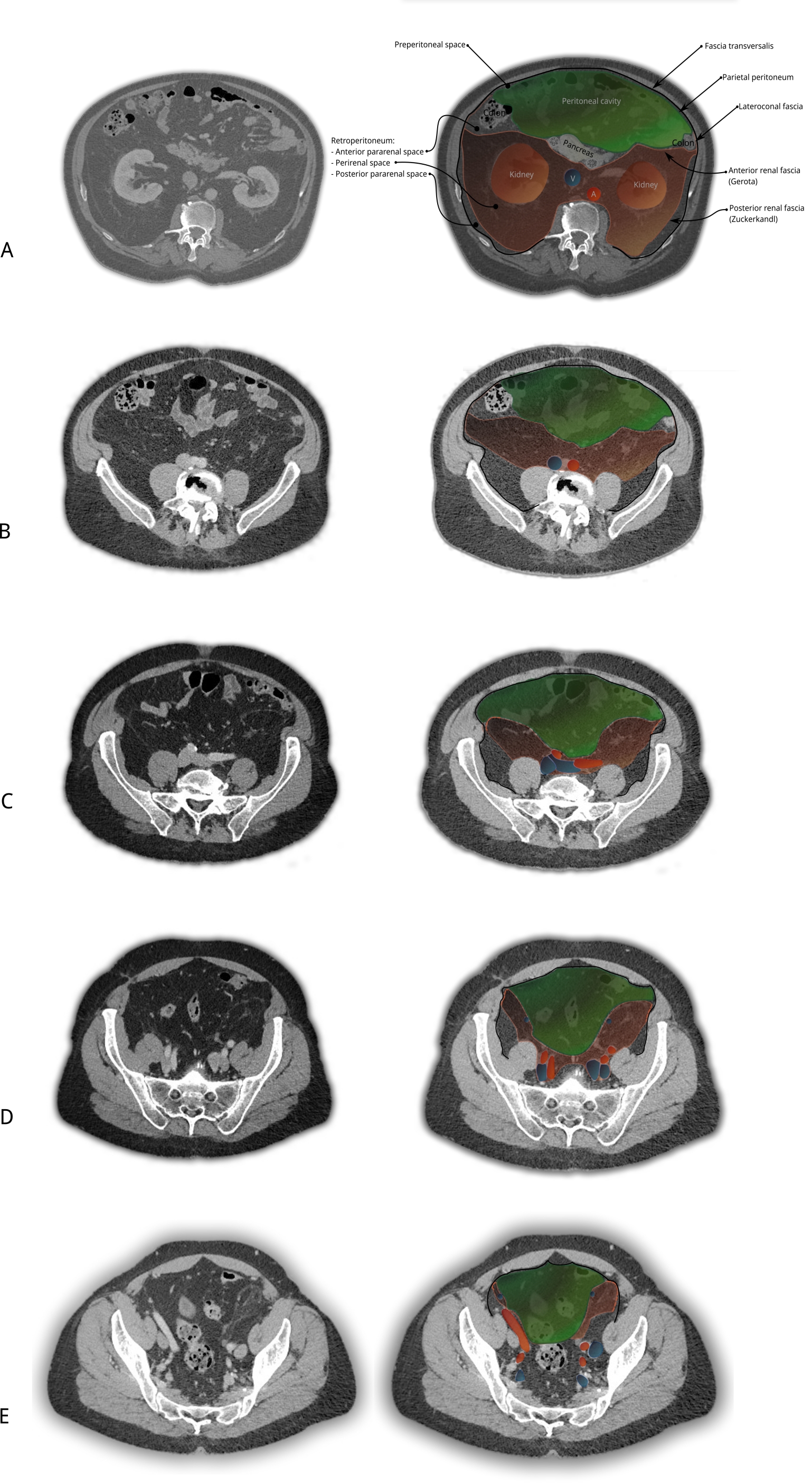


**Supplementary Figure 1.** CT imaging with transverse sections through the abdomen at the level of the kidneys (A), the level of above the umbilicus (B), below the umbilicus (C), anterior superior iliac spine (D) and pelvis at the apex of the urinary bladder (E). The perirenal space and fascia are marked in red, the peritoneal cavity in green. The transversalis fascia is marked black. V abbreviates Vena cava, A abbreviates aorta. Note the contact of the perirenal fascia to to the transversalis fascia laterally.
